# Seed germination ecology of *Bidens pilosa* and its implications for weed management

**DOI:** 10.1038/s41598-019-52620-9

**Published:** 2019-11-05

**Authors:** Bhagirath Singh Chauhan, Hafiz Haider Ali, Singarayer Florentine

**Affiliations:** 1 The Centre for Crop Science, Queensland Alliance for Agriculture and Food Innovation (QAAFI), The University of Queensland, Gatton, Queensland 4343 Australia; 2 Department of Agronomy, University College of Agriculture, University of Sargodha, Sargodha, 40100 Punjab Pakistan; 3 Centre for Environmental Management, Faculty of Science and Technology, Federation University Australia, Ballarat, Victoria 3353 Australia

**Keywords:** Population dynamics, Plant ecology

## Abstract

It is now widely recognized that *Bidens pilosa* has become a problematic broadleaf weed in many ecosystems across the world and, particularly in the light of recent climate change conditions, closer management strategies are required to curtail its impact on agricultural cropping. In this investigation, experiments were conducted to evaluate the effect of environmental factors on the germination and emergence of *B. pilosa*, and also on the response of this weed to commonly available post-emergence herbicides in Australia. The environmental factors of particular interest to this current work were the effect of light and temperature, salinity, burial depth and moisture on *B. pilosa* since these are key management issues in Australian agriculture. In addition, the effects of a number of commonly used herbicides were examined, because of concerns regarding emerging herbicide resistance. In the tested light/dark regimes, germination was found to be higher at fluctuating day/night temperatures of 25/15 °C and 30/20 °C (92–93%) than at 35/25 °C (79%), whilst across the different temperature ranges, germination was higher in the light/dark regime (79–93%) than in complete darkness (22–38%). The standard five-minute temperature pretreatment required for 50% inhibition of maximum germination was found to be 160 °C, and it was further shown that no seeds germinated at temperatures higher than 240 °C. With regard to salinity, some *B. pilosa* seeds germinated (3%) in 200 mM sodium chloride (NaCl) but all failed to germinate at 250 mM NaCl. Germination declined from 89% to 2% as the external osmotic potential decreased from 0 to −0.6 MPa, and germination ceased at −0.8 MPa. Seeding emergence of *B. pilosa* was maximum (71%) for seeds placed on the soil surface and it was found that no seedlings emerged from a depth of 8 cm or greater. A depth of 3.75 cm was required to inhibit the seeds to 50% of the maximum emergence. In this study, application of glufosinate, glyphosate and paraquat provided commercially acceptable control levels (generally accepted as >90%) when applied at the four-leaf stage of *B. pilosa*. However, none of the herbicide treatments involved in this study provided this level of control when applied at the six-leaf stage. In summary, *B. pilosa* germination has been clearly shown to be stimulated by light and thus its emergence was greatest from the soil surface. This suggests that infestation from this weed will remain as a problem in no-till conservation agriculture systems, the use of which is increasing now throughout the world. It is intended that information generated from this study be used to develop more effective integrated management programs for *B. pilosa* and similar weeds in commercial agricultural environments which are tending toward conservation approaches.

## Introduction

*Bidens pilosa*, a member of the Asteraceae family, is a C_3_ annual broadleaf species with a life cycle of 150–360 days^1^. It is thought to have originated in tropical America, but it has now spread aggressively throughout similar climatic regions of the world^2,3^, consequently becoming a troublesome weed in both field and plantation crops. Whilst it is commonly found in gardens, along roadsides, fence rows and in open waste places, of particular interest to this study is that it has been reported to be now a common weed in no less than 31 different crops in more than 40 countries^2^. Some of the crops in which *B. pilosa* has been found as a principal weed are corn, sugarcane, sorghum, cotton, rice, vegetables, and pastures, all of which are critically important to many national economies^4^.

The fruits of *B. pilosa* are sparsely bristled with two to four barbed awns, which facilitate its unintended spread by workers, animals and prevailing winds^2^. As an indication of the serious nature of this problem, it is noted that a single isolated plant of this weed can produce over 30,000 seeds^1^. It also displays two flowering events during the growing season and, of these, the early flowering plants have been claimed to be crucial to its spread in dry seasons^5^ by ensuring that at least some plants will always produce seeds for the next growing season. A previous study reported that *B. pilosa* plants produce two kinds of seeds: long seeds and short seeds^6^. Long seeds were found to germinate more readily than short seeds. The authors also observed that more short seeds are produced under long day conditions, suggesting that the fraction of long and short seeds changes with the season. Furthermore, it has been ascertained that *B. pilosa* commonly serves as a host for fungi, viruses and nematodes, which increases its damaging effect on those crops within which it has become established^2^.

Globally, there are a significant number of studies focusing on the effects of *B. pilosa* in cropping environments^7,8^, suggest that *B. pilosa* is becoming widely recognized as a major impediment to economic food production, but the foci on peculiar geographical situations and the recent effects of climate change issues have strongly suggested that specific Australian studies be carried out. It is therefore expected that, with this new information, a better understanding of the seed germination ecology of *B. pilosa* could be used to develop cultural management programs in a wide range of agricultural concerns, particularly in the light of management strategies which are eschewing the over-use of herbicides.

It is recognized that outcomes of previous studies have shown that seed germination of *B. pilosa* is affected by several environmental factors^9^. Light, for example, has been clearly established as being an important environmental factor for germination^10^, and it is well recognized, for management purposes, that weed seeds requiring light for germination do not emerge when buried deep in the soil. Conversely, for no-till cropping approaches, residual weed seeds are not significantly buried during crop preparation, and thus have ample opportunity to germinate. It has also been established that temperature is an important environmental factor affecting weed seed germination^9^, and it is noted that whilst some weed seeds germinate readily over a wide range of temperatures, others require a specific temperature range. In addition, some seeds are sensitive to pre-emergent temperatures, and knowledge of this also has important implications for weed management. As such, this information needs to be part of any containment process. For example, the narrow windrow burning technique is one of the common systems of harvest weed seed control (HWSC) in Australia^11^. In this technique, chaff and plant residues containing weed seeds are concentrated in a narrow windrow during crop harvest. Later, the chaff is burnt and the high temperature generated from burning destroys much of the weed seed bank on the soil surface. However, it is known that the temperature required to kill weed seeds differs considerably across common problematic weed species, and, currently, such information is not available on *B. pilosa*. Further, it is known that varying moisture conditions and salinity can also have important effects on weed seed germination, and across the Australian continent there is a wide range of conditions that would need to be carefully considered during crop management strategies.

Of increasing concern is that, in fallow situations, where cropping areas are being prepared for sowing, surface weeds, including *B. pilosa*, are commonly controlled using glyphosate and paraquat. However, it is known that herbicide efficacy is significantly reduced if applied to many varieties of large weedy plants, but such information is very limited in the case of *B. pilosa*, especially in ambient Australian conditions. In addition, it is becoming clear that reliance on one or two herbicides over a considerable time period may result in the unintended evolution of herbicide resistance in these weedy species. In this respect, biotypes of *B. pilosa* have already been reported as being resistant to several herbicides, including glyphosate and paraquat^12,13^. In Brazil, acetolactate synthase (ALS)-inhibiting herbicide-resistant biotypes have also been reported^14^. This is a concerning development and strongly suggests that there is a pressing need to evaluate the efficacy of a range of herbicides at different growth stages of *B. pilosa* in order that a more ecologically responsible approach to herbicide use can be developed.

It is noted that some useful information about the germination requirements of *B. pilosa* is available for populations in the USA^7^, but no similar information is available for specifically Australian populations. The concern here is that the previous study in the USA was conducted more than 20 years ago and there is a significant possibility that germination requirements of *B. pilosa* in Australia are different from those of *B. pilosa* in the USA. In addition, very limited information is currently available in the literature globally on the efficacy of a range of herbicides on *B. pilosa*, leaving a gap in the possibility of varying treatments in a specific area to avoid herbicide-resistant evolution.

Therefore, in this study, experiments were conducted in order to build a more detailed knowledge of the biology of *B. pilosa*. In particular, work has been done to determine the effects of temperature, light, salt stress, osmotic stress, and seed burial depth on the germination and emergence of seeds of this species, and to evaluate the relative responses of this weed to a range of commonly available post-emergence herbicides in Australia.

## Results and Discussion

### Effect of temperature and light on germination rate

The role of temperature in the germination of weed species is a complex issue. This observation that fluctuating temperature is a significant factor affecting weed seed germination has been previously recorded. For example, *Eclipta prostrata* (L.) L. has been shown to germinate over a range of temperatures^15^, whereas other species require critical levels of temperatures before they germinate. In the present study, seeds germinated at all the tested temperature ranges, which normally occur during the spring-summer seasons in Queensland, Australia. Our experiments indicated that, in the light/dark regime, germination of *B. pilosa* was higher at alternating day/night temperatures of 25/15 °C and 30/20 °C (92–93%) than at 35/25 °C (79%). These results suggest that *B. pilosa* can emerge throughout the spring and summer seasons in Australia and may also germinate in the early autumn. As an indication of the complexity of this issue, in a previous study carried out in the USA, *B. pilosa* incubated at temperatures ranging from 25/20 to 35/30 °C had maximum germination^7^, and in that study, only a 5 °C difference was maintained between the fluctuating temperatures. However, in the northern regions of Australia, temperatures can fluctuate more than 10 °C between day and night, therefore, this difference of 10 °C was selected between the fluctuating temperatures in the present study. A previous study from South Africa reported that the optimum temperature for germination was 25 °C, which is similar to the temperature (30/20 °C) found in our study^6^.

**Figure 1:**
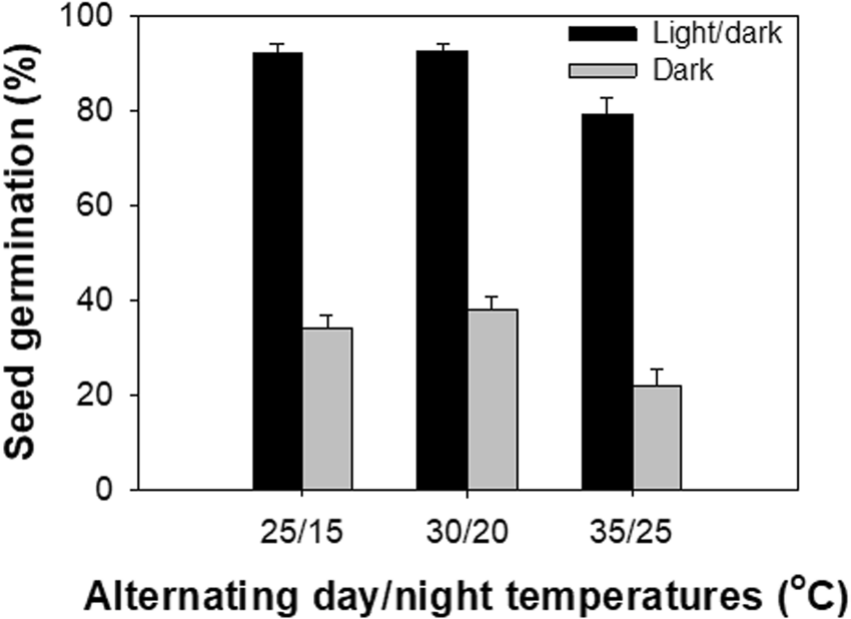
Effect of alternating day/night temperatures (25/15 °C, 30/20 °C, and 35/25 °C) and light (light/dark and dark regimes) on seed germination of *Bidens pilosa*. The vertical bars represent the standard errors of the mean.

Whilst it was found that light was not a prerequisite for germination, it did significantly stimulate germination. This was shown by results which indicated across different temperatures, germination was higher in the light/dark regime (79–93%) than in complete darkness (22–38%) (Fig. 1). It is generally known that light plays an important role in affecting weed seed germination, but germination responses to light vary from species to species. Seeds of some species (e.g., *E. prostrata*) have an absolute requirement of light for germination^15^ and others can germinate in both light and dark conditions^16^. Whilst in this study, *B. pilosa* germinated in both light regimes, seed germination under a 12 h photoperiod was higher than under continuous darkness. In previous studies, it was found that light had a stimulatory effect on *B. pilosa* germination^7,8^.

The results of the present study suggest that *B. pilosa* emergence in the field may be encouraged by reduced or no-till systems, in which seeds are exposed to light and thus a large proportion of weed seeds remain on or close to the soil surface^17^. However, it was observed that a proportion of seeds germinated in darkness, thus *B. pilosa* has the potential of (somewhat reduced) germination even when buried in the soil.

### Effect of pretreatment high temperature on germination

**Figure 2:**
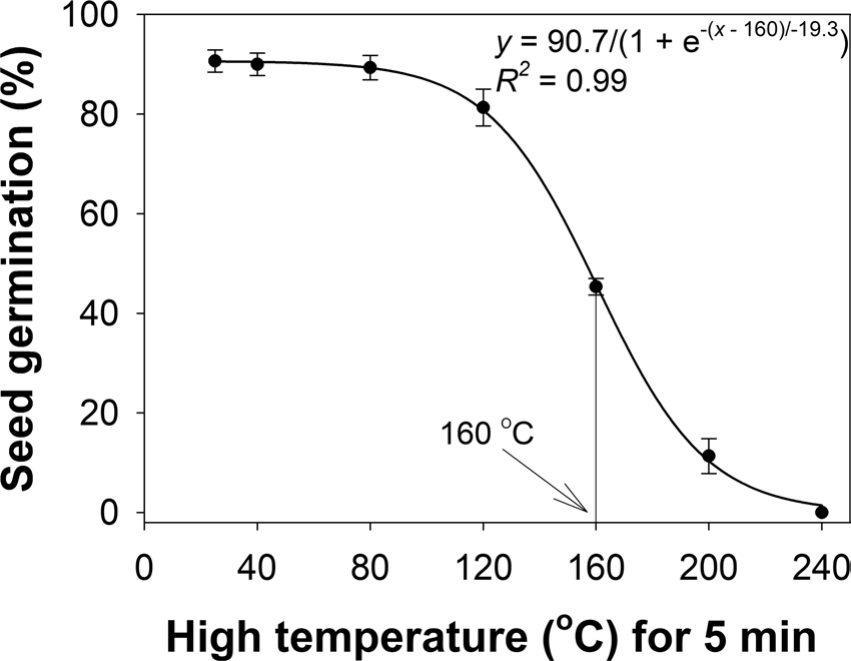
Effect of oven temperature (pre-treatment) for 5 min on seed germination of *Bidens pilosa* when incubated at 30/20 °C in the light/dark regime for 14 days. The line shows a three-parameter sigmoid model fitted to the germination values and the arrow shows the temperature required to inhibit 50% of the maximum germination.

A sigmoid response was observed in the germination of *B. pilosa* seeds with increases in pretreatment (for 5 min) high temperature from 20 to 240 °C (Fig. 2). Germination was >80% when exposed to temperatures up to 120 °C for 5 min, but decreased when exposed to higher temperature. The critical temperature (for 5 min) to inhibit 50% germination was 160 °C and no seeds germinated at 240 °C.

It has been claimed that vegetation burning can increase temperature on the soil surface to greater than 500 °C for more than 5 min^18^. These results suggest that, depending on stubble amount, *B. pilosa* seeds on the soil surface should be easily destroyed by narrow windrow burning. These harvest weed seed control (HWSC) systems have now become an essential part of integrated weed management programs in Australia. For example, a previous study on another Asteraceae weed, *E. prostrata*, reported that it required a temperature of 200 °C for 5 min to completely kill the seeds^15^.

### Effect of salt stress on germination

**Figure 3:**
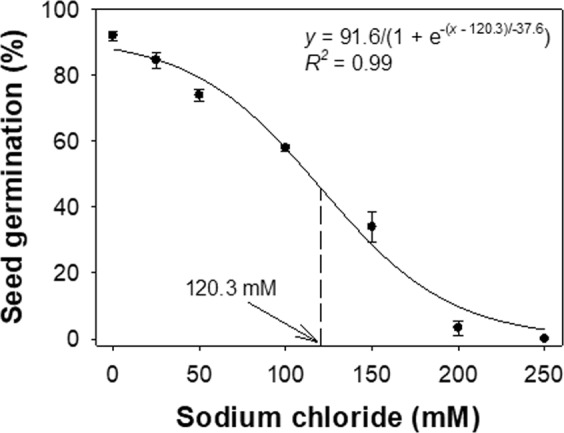
Effect of sodium chloride concentrations on seed germination of *Bidens pilosa*. The line shows a three-parameter sigmoid model fitted to the germination values and the arrow shows the concentration required to inhibit 50% of the maximum germination.

Salinity is a major natural resource issue throughout Australia and is particularly so in southern Australia. Soils with 40 to 100 mM NaCl are considered to have ‘moderate’ salt content and soils with higher levels of NaCl are referred to as ‘high’ salt content^19^. In these investigations, a sigmoid response was observed in the germination of *B. pilosa* seeds with increases in NaCl concentrations from 0 to 250 mM (Fig. 3). The germination was at a maximum (92%) when seeds were incubated in no-stress conditions. Whilst seeds did not germinate at 250 mM NaCl, more than 3% seeds still germinated at 200 mM NaCl. A concentration of 120 mM NaCl inhibited 50% germination.

Similar to *B. pilosa*, seeds of another Asteraceae weed (*E. prostrata*) germinated at high salt concentrations^15^. In contrast, the previous study in the USA reported no germination of *B. pilosa* at 200 mM NaCl, and that study suggested that the weed germination was sensitive to salt stress. Of particular concern in this regard is that in Queensland, while the area of saline land was 48,000 hectares in 2001, it had increased to 107,000 ha by 2002^20^. The relevance of the results of the present study is that they suggest that *B. pilosa* can aggressively germinate in such soil conditions, therefore crop production may be affected in the future by both soil salinity increases and weed competition.

### Effect of water stress on germination

It is widely recognized that, in Australian agricultural areas, water variability, causing levels of water stress, is an increasing phenomenon. An increase in drought conditions characteristic of climate change is beginning to make areas of traditional agriculture unprofitable because the response to water stress conditions, and its management, are not yet well understood.

**Figure 4:**
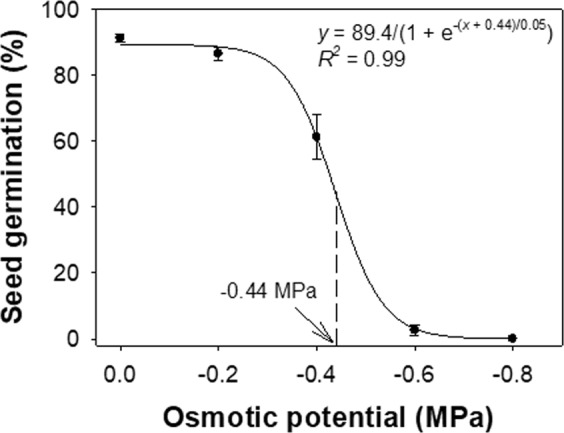
Effect of osmotic potential on seed germination of *Bidens pilosa*. The line shows a three-parameter sigmoid model fitted to the germination values and the arrow shows the osmotic potential required to inhibit 50% of the maximum germination.

In this investigations, a sigmoid response was observed in the germination of *B. pilosa* seeds with decreases in osmotic potential from 0 to −0.8 MPa (Fig. 4). Germination declined from 89% to 2% as osmotic potential decreased from 0 to −0.6 MPa. Germination was completely inhibited at an osmotic potential of −0.8 MPa and an osmotic potential of −0.44 MPa inhibited 50% germination. These results suggest that *B. pilosa* is a weed favored by a moist environment, which resonates with results from the previous study in the USA which indicated that *B. pilosa* was sensitive to low water potential^7^. This finding concurs with the positive association between this species and moist environments which was reported previously^2^. An outcome of this work suggests that a lack of moisture may be the overriding control factor for seed germination in dry soil conditions^21^ since seeds germinate when the water content of the embryo is sufficient to support biochemical processes that lead to cell expansion. Although the effect of flooding (excess soil moisture) was not evaluated in our study, a previous study suggested that seedling emergence of *B. pilosa* is greatly reduced when flooding is maintained^7^. These results suggest that this species may not persist in flooded areas.

### Effect of seed burial depth on emergence

**Figure 5:**
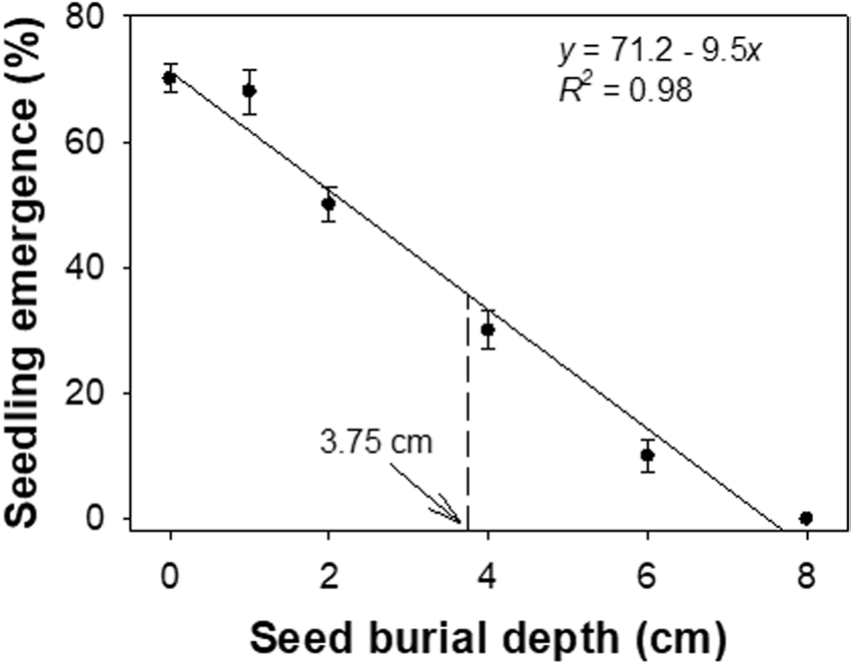
Effect of seed burial depth on seedling emergence of *Bidens pilosa*. The line shows a linear model fitted to the emergence values and the arrow shows the depth required to inhibit 50% of the maximum emergence.

It was clear that *Bidens pilosa* emergence is greatly affected by seed burial depth which is consistent with the positive effect of light on germination. From previous studies, it is known that increased seed burial depth is known to reduce weed seedling emergence^22^ because light can only penetrate minimally into the top soil layer^23^. Our results showed that seedling emergence declined linearly with increased burial depth (Fig. 5), with germination being greatest (71%) for seeds placed on the soil surface. As burial depth increased, seedling emergence declined by 10% for every centimeter increase in immersion in the soil. No seedlings emerged from a depth of 8 cm or greater, and the burial depth required for 50% inhibition of the maximum emergence was 3.75 cm.

Although *Bidens pilosa* germination was maximum on the soil surface, up to 31% of seeds germinated in darkness. It is thought, therefore, that additional factors might be responsible for the (albeit limited) emergence of deeply buried seeds. While light stimulation clearly has an important role in seed germination, and its absence will have a noticeable retarding effect, it is possible that insufficient seed reserves (due to the small size of the seeds) to support seedling emergence could be another factor for low emergence from increased soil depths.

Notwithstanding the incomplete picture of seed emergence parameters, the greater level of surface germination strongly suggests that no-till or conservation agriculture practices would enhance *B. pilosa* seedling emergence. This is because, in such farming systems, a large fraction of weed seeds dropped by previous infestations remain on or close to the soil surface^17^. These results are entirely consistent with the previous study conducted in the USA^7^, in which results were related with observed severe infestations of *B. pilosa* in open undisturbed areas such as citrus row middles.

With regard to possible management strategies, two issues should be noted. First, weed seeds which are present on the soil surface are prone to environmentally facilitated death and from predation by insects. Thus there may be a useful mitigating effect on *B. pilosa* weed infestation where environmental interaction is severe enough or where natural seed predators are common. Second, the physical buildup of *B. pilosa* seeds on the soil surface can be managed by a deep tillage operation that buries *B. pilosa* and other weed seeds below their maximum depths of emergence (8 cm or deeper in the case of *B. pilosa*).

### Effect of herbicides on survival and biomass

Currently, and in the recent past, selective and non-selective post-emergence herbicides have been a major tool to control *B. pilosa* both in productive crops and in fallow conditions, However, it is being recognised that overuse of a particular herbicide can lead to developed resistance by weed species, and also to unanticipated environmental effects in surrounding areas. In this work, a number of herbicides was evaluated in order to ascertain whether a more sensitive and efficient herbicide management approach might be possible to control *B. pilosa*.

It was found that, at the four-leaf stage of emergence, no plants of *B. pilosa* survived following the application of paraquat, and only 3% plants survived following the application of glufosinate (Table 1). Subsequently, application of these treatments at the six-leaf stage resulted in 18% and 38% surviving plants, respectively. Less than 20% plants survived following the application of 2,4-D or 2,4-D plus picloram at the four-leaf stage; however, the number of surviving plants increased to greater than 50% when these herbicides were applied at the six-leaf stage. In these experiments, fluroxypyr and saflufenacil were the poorest herbicide treatments, with 97–100% of plants surviving the treatment, irrespective of their leaf stage.

**Table 1:** Effect of post-emergence herbicides on seedling survival, shoot biomass, and control of *Bidens pilosa* when applied at the four- and six-leaf developmental stages.

Herbicide	Rate (g ha^−1^)	Four-leaf stage			Six-leaf stage		
		Survival (%)	Biomass (g/plant)	Control (%)	Survival (%)	Biomass (g/plant)	Control (%)
Control	0	0	0.155	0	100	0.244	0
2,4-D	1050	17	0.031	80	64	0.116	52
2,4-D + picloram	375	15	0.016	90	58	0.143	41
Fluroxypyr	100	97	0.070	55	100	0.160	34
Glufosinate	750	3	0.002	99	18	0.050	80
Glyphosate	675	48	0.011	93	100	0.104	57
Paraquat	500	00	0.000	100	38	0.155	36
Saflufenacil*	12	100	0.057	63	100	0.122	50
LSD		19.5	0.030		16.8	0.100	

^*^Hasten at 1% was added to saflufenacil.

Our shoot biomass data suggested that glufosinate, glyphosate and paraquat provided greater than 90% control when applied at the four-leaf stage. However, none of these herbicide treatments provided greater than 80% control when applied at the six-leaf stage, and, regardless of growth stage, other herbicide treatments did not provide commercially acceptable control (>90%) of *B. pilosa*.

This study found that none of the selective herbicides (for example, 2,4-D) provided commercially accepted control of this weed, which suggests that growers will face significant challenges for controlling *B. pilosa* in herbicide-resistant cereals. To achieve any reasonable economic level of control, it appears that they would need to integrate herbicides such as 2,4-D with other cultural tools to control *B. pilosa*.

Non-selective post-emergence herbicides (glufosinate, glyphosate and paraquat) provided >90% control but only when applied at the four-leaf stage, suggesting that *B. pilosa* could be controlled effectively in fallows or before crop planting provided that these herbicides are applied at or before the four-leaf stage. Generally, herbicide efficacy is reduced when applied on large plants^24^ and therefore, their rates may need to be increased to achieve effective control of large plants. In the absence of effective herbicides for controlling large *B. pilosa* plants, protoporphyrinogen oxidase (PPO) and photosystem II inhibitors should be evaluated for *B. pilosa* control. PPO herbicides inhibit the PPO enzyme which disrupts photosynthesis and causes membrane disruption and plant death^25^. Photosystem II inhibitors interfere with the photosynthesis process by which plants fix energy from sunlight^25^.

Saflufenacil is a relatively new herbicide in Australia and it has been found effective on other Asteraceae species, such as *Conyza canadensis*^26^. In our study, this herbicide provided poor control of *B. pilosa*; however, saflufenacil is not registered to control *B. pilosa* in Australia. This tolerance might be due to its ability to rapidly metabolize and detoxify the herbicide.

Plants in our study were grown in pots in a screen house, where they received conducive growing conditions. Plants grown in the field condition may be stressed and of variable sizes, which may reduce herbicide efficacy further. Therefore, there is a need to evaluate herbicides in the field conditions.

### Summary

As these results have shown that *B. pilosa* germinates over a wide range of spring and summer temperatures, this suggests that the weed will germinate throughout the spring and summer seasons in the northern region of Australia. The robust nature of this species was also demonstrated by the observation that whilst light greatly stimulated seed germination of *B. pilosa*, a significant number of seeds also germinated in the dark. In addition, *B. pilosa* was found to germinate at high salt levels, but, in common with many other species, preferred a moist environment.

Based on available evidence, it is suspected that tillage operations that bury seeds deeper than 8 cm would limit *B. pilosa* seedling emergence, and that no-till or reduced-till systems, which do not cover seeds, would be most likely to promote seedling emergence. These results are consistent with observed *B. pilosa* infestations in the northern region of Australia, where no-till is the dominant farming system. The temperature investigations suggest that to support the no-till farming system, windrow burning after crop harvest may help in reducing weed infestation by destroying the seed bank on the surface layers of the soil. In addition, with no-till fallow periods, application of the non-selective post-emergence herbicides at or before the four-leaf stage of *B. pilosa* can significantly reduce its density and prevent reseeding in the following season. Knowledge gained from these studies in Australian conditions should be helpful for devising regional control measures in cropping situations and for limiting the future spread of *B. pilosa*. Inferences from the results of this study should be carefully related to the field conditions as this study was conducted in the laboratory and screenhouse, where conditions are different from the field. In the field, results may differ; therefore, there is a need to conduct field trials.

## Material and Methods

### Seed collection and germination test

Seeds of *B. pilosa* were collected during May 2015 from the edges of established soybean and sorghum fields at the research farm of the University of Queensland, Gatton (27°32′S, 152°20′E), Queensland, Australia. As the first author work at the university, no permission was needed to collect the weed seeds, as well as that the studies did not involve endangered or protected species. Seeds were collected from at least 100 plants, dried in a glasshouse for two days, cleaned, and stored in a labelled airtight container at room temperature (20 °C) until used in the experiments.

Aspects of *Bidens pilosa* germination were evaluated by placing experimental lots of 25 seeds in a 9 cm diameter Petri dish containing two layers of Whatman No.1 filter paper moistened with either 5 ml of distilled water or a relevant experimental solution. The dishes were placed in sealed plastic bags (to avoid moisture loss due to evaporation) and then placed in an incubator at fluctuating day/light temperatures of 30/20 °C in light/dark conditions (optimum conditions), unless otherwise indicated. The photoperiod in the incubator was set at 12 h light to coincide with the high-temperature period. In this investigation, seed germination was evaluated up to a maximum of 14 days after the start of the experiment, as no seeds germinated in a preliminary control experiment in the incubator after this period. For the purposes of common reportage, when there was visible protrusion of the radicle, the seed was considered ‘germinated’.

### Effect of temperature and light on germination

The effect of variable temperature and light conditions on the germination of *B. pilosa* was determined by placing seeds in incubators set at three different day/night temperature regimes of 25/15 °C, 30/20 °C, and 35/25 °C in both light/dark and dark regimes. These temperature regimes were selected to reflect the temperature fluctuation during the spring to summer period in Queensland. In the dark regime, the Petri dishes were wrapped in double layers of aluminum foil.

### Effect of ‘pretreatment’ high temperature on germination

The effect of pretreatment high temperature on germination of *B. pilosa* was determined by placing seeds in an oven for 5 min at 40, 80, 120, 160, 200 and 240 °C. The treated seeds were then incubated at the optimum conditions described earlier. Seeds stored at room temperature (20 °C) were used in the control treatment.

### Effect of salt stress on germination

The effect of salt stress^13^ on germination of *B. pilosa* was determined by placing seeds on filter paper in Petri dishes with 5 ml solutions of 0, 25, 50, 100, 150, 200, and 250 mM of sodium chloride (NaCl). These dishes were incubated at the optimum conditions described earlier.

### Effect of water stress on germination

The effect of water stress on germination of *B. pilosa* was determined by placing seeds on filter papers in Petri dishes with 5 ml solution of 0, −0.2, −0.4, −0.6, −0.8, and −1.0 MPa. The solutions were prepared by dissolving polyethylene glycol 8000 in distilled water^27^.

### Effect of seed burial depth on emergence

The effect of seed burial depth on seedling emergence of *B. pilosa* was determined in the screen house weed science facility of the University of Queensland, Gatton, Queensland. Fifty weed seeds were placed on the soil surface in 15-cm diameter plastic pots or covered with the same soil to achieve burial depths of 1, 2, 4, 6, 8, and 10 cm. The soil used in this experiment was red clay and had a pH of 5.5 and an electrical conductivity of 0.6 dS m^−1^. There was no background seedbank of *B. pilosa* in the soil. Initially, an overhead mist sprinkler was used to irrigate pots, but during the experiment the pots were sub-irrigated. Emergence was defined as the first appearance of cotyledons, and the experiment was run until 35 d after seed burial.

### Effect of herbicides on survival and biomass

Fifteen seeds of *B. pilosa* were planted at 0.5 cm depth in 11-cm diameter plastic pots, using a weed-free commercial potting mix. Seedlings were thinned to five plants per pot three days after emergence and sprayed with different herbicide treatments at the four- and six-leaf stages. There were control treatments for each leaf stage in which herbicides were not sprayed. The herbicides and their application rates are given in Table 1. Herbicides were applied using a research track sprayer that delivered 108 L ha^−1^ water at a spray pressure of 200 kPa. Flat nozzles (Teejet 110015) were used in the sprayer. Seedling survival was determined 28 days after herbicide treatment with the criterion of at least one green leaf on the plant indicating survival. Survived plants were cut at the soil surface and dried in an oven at 70 °C for 72 h to measure dry biomass, and the results were expressed as percent control.

### Statistical analyses

All experiments were conducted in a randomized complete block design and each treatment was replicated three times. All experiments were conducted twice and the second run was started within a month of termination of the first experiment. The data variance was visually examined by plotting the residuals to check homogeneity of variance before statistical analysis. ANOVA was performed on the original values, and showed that there were no differences between the two experimental periods. Henceforth, the data from the repeated experiments were combined for analysis.

The data on the temperature and light and the herbicide experiments were subjected to ANOVA, and means were separated using the least significant difference (LSD) test at the 5% level of significance (Gen Stat 18^th^ Edition). For other experiments, regression analysis was carried out using SigmaPlot 13.0 (Systat Software, Inc). Germination data from high temperature, salt and water potential treatments were modelled using a functional three-parameter sigmoid model:

$$Y=Y\,\max \,/\{1+\exp [\,-\,(x-x0)/b]\}$$

In this model, *Y* is the total germination (%) at the NaCl concentration or osmotic potential *x*, *Y*_*max*_ is the maximum germination (%), *x*_0_ is the NaCl concentration or osmotic potential required for 50% inhibition of maximum germination, and *b* indicates the slope. Seedling emergence resulting from different burial depths was modelled using a linear model:

$$Y=a+bx$$

In this model, *Y* is the predicted emergence (%) as a function of burial depth *x*, *a* is the *Y* intercept, and *b* describes the slope of the curve.
